# Validation of Deleterious Mutations in Vorderwald Cattle

**DOI:** 10.1371/journal.pone.0160013

**Published:** 2016-07-29

**Authors:** Sina Reinartz, Ottmar Distl

**Affiliations:** Institute for Animal Breeding and Genetics, University of Veterinary Medicine Hannover, Hannover, Germany; Humboldt-Universitat zu Berlin, GERMANY

## Abstract

In Montbéliarde cattle two candidate mutations on bovine chromosomes 19 and 29 responsible for embryonic lethality have been detected. Montbéliarde bulls have been introduced into Vorderwald cattle to improve milk and fattening performance. Due to the small population size of Vorderwald cattle and the wide use of a few Montbéliarde bulls through artificial insemination, inbreeding on Montbéliarde bulls in later generations was increasing. Therefore, we genotyped an aborted fetus which was inbred on Montbéliarde as well as Vorderwald x Montbéliarde crossbred bulls for both deleterious mutations. The abortion was observed in an experimental herd of Vorderwald cattle. The objectives of the present study were to prove if one or both lethal mutations may be assumed to have caused this abortion and to show whether these deleterious mutations have been introduced into the Vorderwald cattle population through Montbéliarde bulls. The aborted fetus was homozygous for the SLC37A2:g.28879810C>T mutation (ss2019324563) on BTA29 and both parents as well as the paternal and maternal grandsire were heterozygous for this mutation. In addition, the parents and the paternal grandsire were carriers of the MH2-haplotype linked with the T-allele of the SLC37A2:g.28879810C>T mutation. For the SHBG:g.27956790C>T mutation (rs38377500) on BTA19 (MH1), the aborted fetus and its sire were heterozygous. Among all further 341 Vorderwald cattle genotyped we found 27 SLC37A2:g.28879810C>T heterozygous animals resulting in an allele frequency of 0.0396. Among the 120 male Vorderwald cattle, there were 12 heterozygous with an allele frequency of 0.05. The SLC37A2:g.28879810C>T mutation could not be found in further nine cattle breeds nor in Vorderwald cattle with contributions from Ayrshire bulls. In 69 Vorderwald cattle without genes from Montbéliarde bulls the mutated allele of SLC37A2:g.28879810C>T could not be detected. The SHBG:g.27956790C>T mutation appeared unlikely to be responsible for the present case of abortion and, in addition, we observed this mutation in a homozygous state in a living animal. In conclusion, we could demonstrate the first case of an aborted fetus carrying the deleterious SLC37A2:g.28879810C>T mutation homozygous and show that this deleterious mutation had been introduced through Montbéliarde bulls into Vorderwald cattle.

## Introduction

The decrease in fertility and conception rate in dairy cattle is of major concern worldwide [1]. Vorderwald cattle is a local breed with a population size of approximately 8,400 herd book cows and 220 herd book bulls. The main breeding area is the Black Forest in Baden-Württemberg, in the southwest of Germany [2]. Vorderwald are dual purpose cattle well adapted to the harsh conditions of the Black Forest. Anecdotical reports on animals with the typical phenotypic coat colour and body conformation for this cattle breed with its origin in the Black Forest date back as early as 1544 [3]. Vorderwald cattle became an important breed for this region due to the specific properties for a mountainous region and a low to moderate input of concentrates for beef and milk production. In order to increase genetic progress in milk and beef production and to reduce inbreeding, bulls from foreign cattle breeds were introgressed for a limited time period, starting in the 1970s and continuing until the 1990s [2–6]. Four Ayrshire bulls were crossed into the Vorderwald population in the 1970s and in the 1980s, introgression took place with five Red Holstein bulls. In the late 1990s, five Montbéliarde bulls from France were introduced in the Vorderwald population and widely disseminated through artificial insemination (AI) [2]. In the actual Vorderwald population the contribution from Ayrshire bulls is estimated at approximately 5% and from Red Holstein bulls at approximately 10% [4–5]. The largest contributions of approximately 40% to the actual gene pool of the Vorderwald cattle came from Montbéliarde bulls [2–5].

In an experimental herd of Vorderwald cattle at the Institute of Animal Breeding and Genetics of the University of Veterinary Medicine Hannover an abortion was observed. The parents of the aborted fetus were Vorderwald cattle which had French Montbéliarde as well Vorderwald x Montbéliarde crossbred bulls as ancestors. In Montbéliarde bulls two recessive lethal haplotypes (MH1 and MH2) harbouring candidate causative mutations were detected [7]. A significant decrease of the conception rate was observed for matings among Montbéliarde carrier bulls and daughters descending from these bulls. For MH1 located on the bovine chromosome (BTA) 19, a strong candidate mutation was identified in the *sex-hormone binding globulin* (*SHBG*) gene. This mutation (g.27956790C>T, rs38377500) is predicted to cause a premature stop codon (p.Q52X) in *SHBG*. The *solute carrier family 37 member 2* (*SLC37A2*) gene was the strongest MH2 candidate. This mutation (g.28879810C>T, ss2019324563) was also predicted to introduce a premature stop codon (p.R12X). Other mutated genes were not identified within the MH2 haplotype.

The objective of this work was to test if the abortion of this Vorderwald x Montbéliarde crossbred fetus may be caused by one or both of these presumed lethal mutations because inspection of the pedigree showed inbreeding on Montbéliarde bulls. With the ability to observe a lethal recessive homozygous mutation in an aborted fetus may give support for a direct evidence of the lethality for the first time. Further validation of both recessive lethal mutations was performed in random samples of Vorderwald cattle including further relatives of the parents of the aborted fetus and Vorderwald cattle distantly related to the aborted fetus. Genotyping of Vorderwald bulls used in AI should show whether these lethal mutations may have been disseminated across Vorderwald herds through AI. Vorderwald cattle without Montbéliarde bulls in their pedigrees should serve as controls for the origin of these lethal mutations in Montbéliarde.

## Results and Discussion

### Phenotype

**Fig 1:**
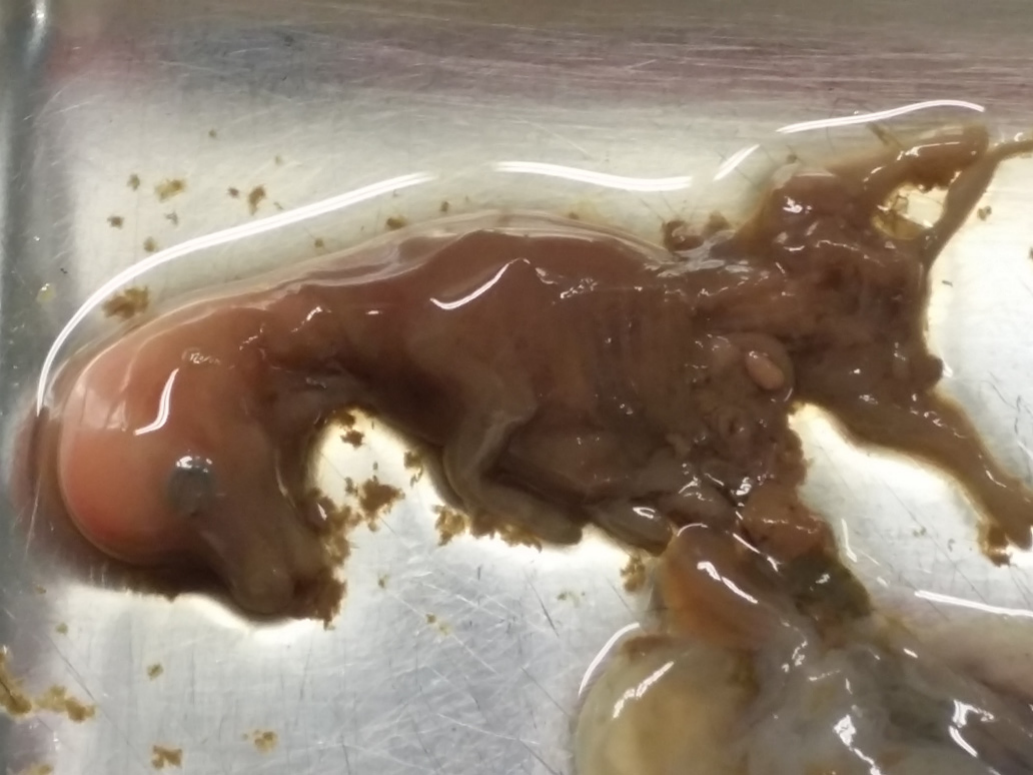
Aborted fetus observed in the experimental Vorderwald cattle herd. The fetus had a crown-rump length of 70 mm.

In an experimental herd consisting of thirteen female and eight male Vorderwald cattle an abortion after a gestation length of 75 days was observed (Fig 1). Crown-rump length of the fetus was 70 mm indicative for a gestation length of 60–90 days. The experimental herd is free from Schmallenberg virus, bovine herpes virus type 1 (BHV_1_) and bovine viral diarrhoea (BVD) virus as well as antibodies against Schmallenberg and BVD virus could not be detected, and thus, these viral agents could not have caused the abortion. We could not find obvious morphological changes of the fetus or histopathological changes of the organs.

### Validation study

**Fig 2:**
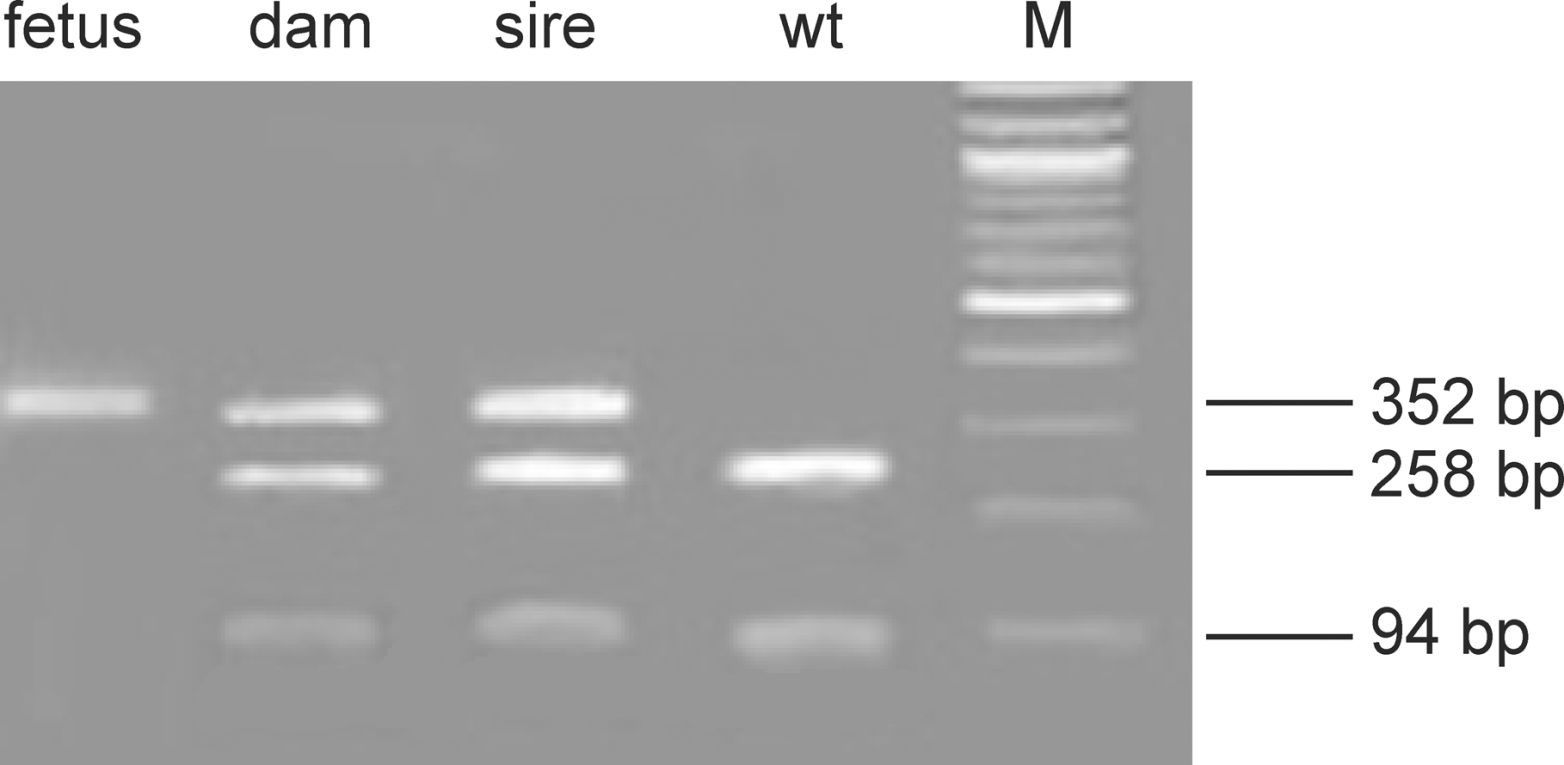
PCR-based restriction fragment analysis of the SLC37A2:g.28879810C>T mutation for the aborted fetus and its sire and dam. The amplicon with the mutated allele has a size of 352 base pairs (bp). The amplicon for the wildtype is digested by the restriction enzyme KpnI in fragments with sizes of 94 and 258 bp. The fetus homozygous for the mutated allele, the heterozygous dam and sire and an unrelated animal homozygous for the wildtype allele are shown.

**Fig 3:**
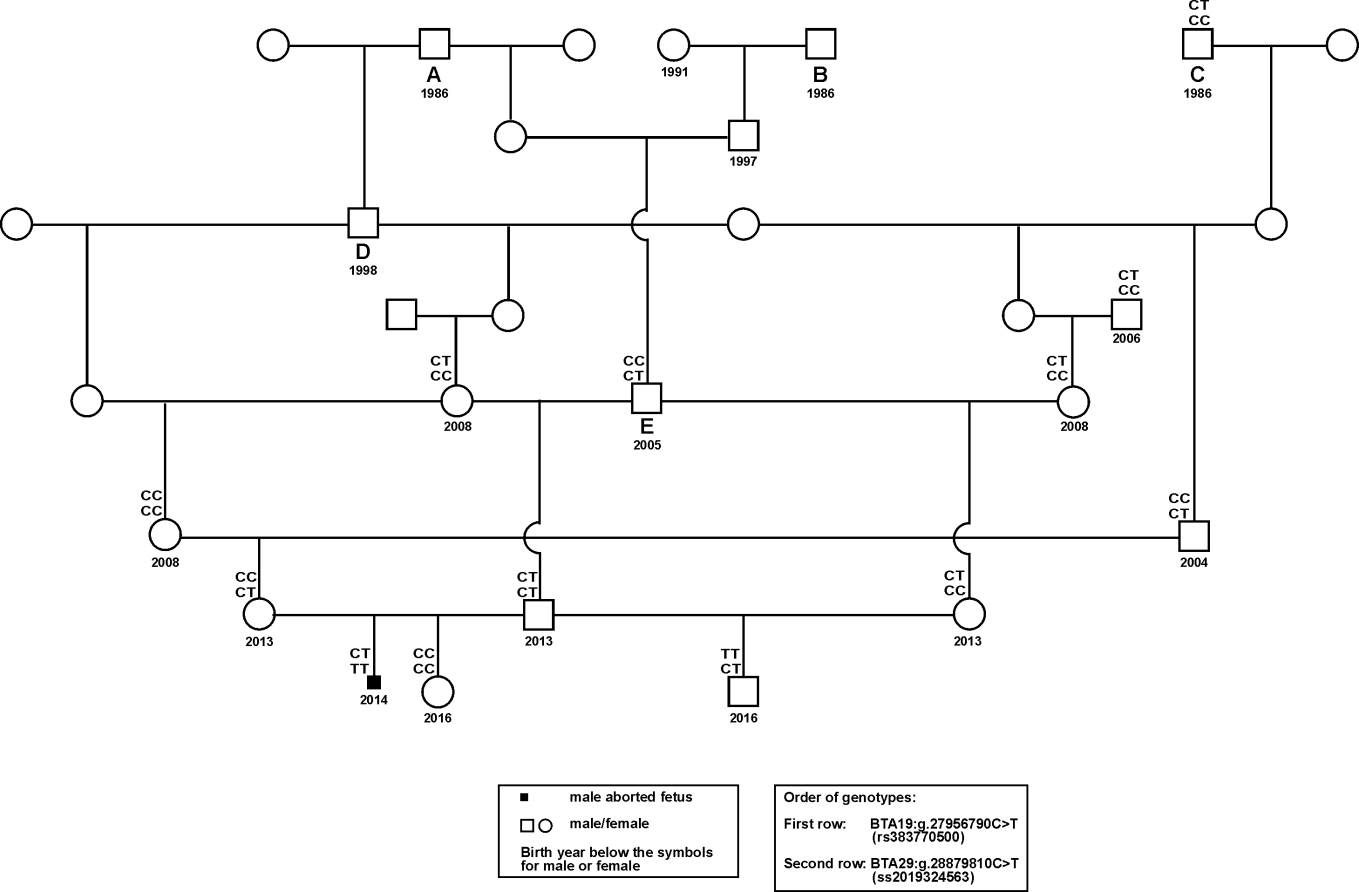
Pedigree of Vorderwald cattle with the aborted fetus and genotypes for the single nucleotide variant (SNV) SHBG:g.27956790C>T (rs38377500) located on bovine chromosome (BTA) 19 and the SNV SLC37A2:g.28879810C>T (ss2019324563) located on BTA29. Ancestors A, B and C are French Montbéliarde bulls. Ancestor A may be the likely source of the SLC37A2:g.28879810C>T mutation for the aborted fetus. The mutated allele was transmitted by the paternal and maternal grandsire to the parents of the aborted fetus.

The aborted fetus was homozygous for the SNV SLC37A2:g.28879810C>T on BTA29 (MH2) and both parents were heterozygous (Fig 2) as well as the paternal and maternal grandsire were heterozygous (Fig 3). Thus, the present analysis could demonstrate the first case of an aborted fetus showing a likely homozygous lethal mutant. Inspection of the pedigree of the aborted fetus revealed inbreeding on male ancestors. These common ancestors were Vorderwald x Montbéliarde crossbred bulls (D, E) and a French Montbéliarde (A).

As a homozygous state of the lethal mutation could only be due to inbreeding on Vorderwald bulls upgraded with Montbéliarde bulls, we calculated the proportion of Montbéliarde blood and the inbreeding coefficients for the aborted fetus using the software OPTI-MATE, version 3.88 [8]. The aborted fetus had an inbreeding coefficient of 10.34%. Inbreeding was mainly caused by Vorderwald x Montbéliarde crossbred bulls (D, E) and a French Montbéliarde founder bull (A). The highest contributions to the inbreeding coefficient of the aborted fetus came from the bulls E (60.7%), D (22.7%) and A (13.2%). Among all further 341 Vorderwald cattle genotyped for the SLC37A2:g.28879810C>T SNV, we found 27 heterozygous animals resulting in an allele frequency of 0.0396 (Table 1). However, the deleterious *SLC37A2* mutation could not be identified in a homozygous state in a living animal from our random sample of Vorderwald cattle. We repeated the same mating among the parents with the observed abortion. A normal healthy female calf was born in 2016 and this calf had the homozygous wildtype genotype. A further normal male paternal half-brother born in 2016 was heterozygous for the SLC37A2:g.28879810C>T SNV. Test for Hardy-Weinberg equilibrium was not significant (P>0.05) employing the ALLELE procedure of SAS/Genetics, version 9.4 (Statistical Analysis System, Cary NC, USA). All 69 Vorderwald cattle with no introgression of Montbéliarde genes according to the pedigree analysis were homozygous wildtype. The oldest SLC37A2:g.28879810C>T heterozygous animal was born in 2004 and an increasing frequency of heterozygous animals was obvious for the following birth years. In addition, we could not find this SNV in 49 Vorderwald cattle born before 2004. Thus, it is likely that the deleterious *SLC37A2* mutation had been introgressed through Montbéliarde bulls.

**Table 1:** Number of Vorderwald cattle and their frequency of heterozygous (het) and homozygous (hom) mutated genotypes for the mutations SHBG:g.27956790C>T (rs38377500) located on bovine chromosome (BTA) 19 and SLC37A2:g.28879810C>T (ss2019324563) located on BTA29 (UMD3.1) for the samples from the experimental herd, the aborted fetus and farms in the Black Forest.

Origin of the	N	SHBG:g.27956790C>T		SLC37A2:g.28879810C>T	
samples		(rs38377500)		(ss2019324563)	
		het	hom	het	hom
Experimental herd	21	4	1	3	0
Aborted fetus	1	1	0	0	1
Farms	320	70	1	24	0
Total	342	75	2	27	1

We tested the paternal grandparents and the parents of the aborted fetus and its paternal half-brother and the French Montbéliarde bull (animal C) from the available pedigree for presence of the MH2 haplotype [7]. We could demonstrate a unique haplotype associated with the T-allele of the SLC37A2:g.28879810C>T SNV spanning from 28,523,337 to 28,908,928 bp on BTA29 and corresponding exactly with the French Montbéliarde haplotype previously reported [7] (S1 Document). This haplotype did not occur in other animals genotyped, particularly not in the French Montbéliarde bull (animal C).

To test the hypothesis of a potential influence on Ayrshire bulls being introgressed prior to Montbéliarde bulls, we also calculated the proportion of Ayrshire blood. The aborted fetus had no contribution from Ayrshire bulls and the same was found for all 27 Vorderwald cattle heterozygous for the SLC37A2:g.28879810C>T SNV. All other Vorderwald cattle with genetic contributions from Ayrshire bulls up to 13% did not carry the SLC37A2:g.28879810C>T SNV.

The proportion of Montbéliarde blood was significantly higher in heterozygous animals compared to homozygous wildtype animals (0.36 versus 0.24, P = 0.0016). The introgression of Montbéliarde bulls into Vorderwald cattle and their heavy use in AI spread the deleterious *SLC37A2* mutation in the crossbred population of Vorderwald cattle. No significant deviation in the allele and heterozygote frequencies was found among sexes. Among the 119 Vorderwald males, there were 11 heterozygous. The allele frequency in males was 0.0462 and in females 0.0341.

In order to validate the origin of the *SLC37A2* SNV in Montbéliarde cattle, we genotyped 96 German Fleckvieh and 105 animals from further nine cattle breeds including Angus, Blonde d’Aquitaine, Charolais, German Brown, German Yellow, Holstein, Limousin, Limpurger and Salers. In neither of these breeds, we could detect the *SLC37A2* SNV what is indicative for a private mutation in Montbéliarde cattle.

The MH2-associated mutation within the *SLC37A2* gene has been proposed to lead to a lack in this solute carrier protein [7]. SLC37A2 has been identified as an endoplasmic reticulum (ER)-associated phosphate-linked, glucose-6-phosphate (G6P) antiporter [9–10] having a key role in cellular energy metabolism [11–14]. Severe deficiency in the enzymes G6PD1, GPI1, GCK being involved in G6P metabolism was shown to lead to embryonic lethality in mouse [11–13] and G6PD deficiency to defective embryogenesis in *Caenorhabditis elegans* [14]. The SLC37A2 gene carries a conserved vitamin D receptor (VDR) binding site [15–16]. Vitamin D3 directly effects the expression of *SLC37A2* in hematopoietic cells via its metabolite 1α,25-dihydroxyvitamin D_3_ with a high affinity to the VDR domain of *SLC37A2* [15–16]. Vitamin D_3_ has a well-known role in regulation of calcium and phosphate homeostasisis, but is also involved in cellular growth, intracellular metabolism as well as innate and adaptive immunity [17–18].

Within the genomic region of the MH2-haplotype as identified in our pedigree a total of 12 genes are annotated in the bovine reference genome assembly UMD3.1. Mutations in genes other than *SLC37A2* have not been identified as possibly damaging in 5 Montbéliarde bulls carrying the MH2-haplotype [7] nor one of these genes was found to be associated with reduced fertility, conception rates or abortion in cattle (http://www.animalgenome.org/cgi-bin/QTLdb/BT/index) or in another species after a search using PubMed (http://www.ncbi.nlm.nih.gov/pubmed?cmd=Search). The *SLC37A2* SNV containing MH2-haplotype which is very likely to have been transmitted from Montbéliarde bulls into Vorderwald cattle and the aborted fetus with the homozygous mutant *SLC37A2* SNV support the assumption of a deleterious effect of this *SLC37A2* mutation. On the other hand, a normal fullsib with the homozygous wildtype genotype after a repeated mating with the same parents and a normal paternal halfsib with a heterozygous genotype may be supportive that the deleterious effect is due to the SLC37A2:g.28879810C>T SNV. Other mutations seem unlikely because two closely related healthy calves carrying not the homozygous mutant genotype were delivered after normal length of gestation and not aborted.

For the SHBG:g.27956790C>T (rs383770500) on BTA19 (MH1), the aborted fetus and its sire were heterozygous and the dam homozygous wildtype. The allele frequency for all samples from Vorderwald cattle genotyped was 0.1155 and the number of heterozygous animals was 75 (Table 1). We found one male healthy calf in our experimental herd and one Vorderwald cow on a dairy farm with the homozygous mutated genotype. The male healthy calf with the mutated homozygous genotype is a paternal half-brother to the aborted fetus. In addition, we could find the rs383770500 variant before introgression of Montbéliarde bulls began. This animal was a heterozygous Vorderwald bull born in 1982. Thus, the rs383770500 variant could not be validated as a lethal mutant because two normal animals were homozygous for this mutation and this SNV was present in our samples genotyped before introgression of Montbéliarde bulls into Vorderwald cattle began in the late 1990s. For this SNV, no significant deviation in the allele and heterozygote frequencies from Hardy-Weinberg equilibrium was found. Furthermore, the frequency of the rs383770500 allele was not associated with the genetic contributions of Montbéliarde or Ayrshire bulls. We did also follow up this SNV in 96 German Fleckvieh and could find this *SHBG* SNV in two German Fleckvieh animals. The *SHBG* SNV seems to be a common mutation and not associated with embryonic mortality.

## Conclusion

In conclusion, we could observe the deleterious variant of the *SLC37A2* gene in Vorderwald cattle through the uniquely homozygously mutated genotype of an aborted fetus and genotyping ancestors in the pedigree of this case. Furthermore, it is very likely that the deleterious *SLC37A2* mutation had been introduced through Montbéliarde bulls and inbreeding on bulls heterozygous for this mutation may be causing embryonic or fetal losses in Vorderwald cattle. Other cattle breeds with genetic contributions to Vorderwald cattle such as German Fleckvieh, Red Holstein and Ayrshire were very unlikely to carry this mutation and could be ruled out as carriers for this lethal *SLC37A2* mutation.

## Materials and Methods

### Ethics statement

All animal work has been conducted according to the national and international guidelines for animal welfare. The Lower Saxony state veterinary office at the Niedersächsisches Landesamt für Verbraucherschutz und Lebensmittelsicherheit, Oldenburg, Germany, was the responsible Institutional Animal Care and Use Committee (IACUC) for this specific study. The present study had been specifically approved by this IACUC of Lower Saxony, the state veterinary office Niedersächsisches Landesamt für Verbraucherschutz und Lebensmittelsicherheit, Oldenburg, Germany (registration number 33.9-42502-04-12/1036).

### Animals

An experimental herd consisting of thirteen female and eight male Vorderwald cattle is being kept at the Institute for Animal Breeding and Genetics, University of Veterinary Medicine Hannover. Each animal of this experimental herd has been tested for Schmallenberg virus, BVD virus and bovine herpes virus type 1 (BHV_1_) using ELISA tests (IDEXX Laboratories, Hoofddorp, The Netherlands). In addition, blood samples were tested for Schmallenberg and BVD virus antibodies using ELISA tests (IDEXX Laboratories). The fetus underwent a necropsy and a histopathological examination. A tissue sample from the aborted fetus and EDTA-blood samples were asserved from all Vorderwald cattle of this experimental herd. In addition, EDTA-blood, hair root or sperm samples were available from further 320 randomly sampled Vorderwald cattle. Altogether, we collected samples of 220 cows or heifers born between 2000 and 2015, and 119 males born from 1982 to 2014. Out of the 119 males, 78 were bulls employed in AI and the other 41 were bulls in natural service or male calves (Table 1). Pedigree data were recorded for all animals up to ten generations. Parentage of the aborted fetus was validated using the microsatellites MCM42, INRA197, MCM156, BM8125, B42MS, BM6438, CSRD247, B349MS1, MCM136, AGLA269, B269MS1 and B269MS3 and haplotype analysis for the parental alleles.

Further samples from 96 German Fleckvieh and 105 animals from further nine cattle breeds including Angus, Blonde d’Aquitaine, Charolais, German Brown, German Yellow, Holstein, Limousin, Limpurger and Salers were employed from our biobank for validation.

### Genotyping and sequencing

Genomic DNA was isolated using 500 μl EDTA-blood and standard protocols. Genotyping of the single nucleotide variants (SNVs) SHBG:g.27956790C>T (rs383770500) on BTA19 (MH1) and SLC37A2:g.28879810C>T on BTA29 (MH2) was performed using polymerase chain reaction-restriction fragment length polymorphisms (PCR-RFLP) with the restriction enzymes BsmFI (*SHBG*) and KpnI (*SLC37A2*). All positions of the SNVs are based on cattle reference genome assembly UMD3.1. For genotyping the SNV on BTA19, a missmatch PCR was developed (S1 Table). The results of the mismatch PCR for the homozygous mutated cow and its heterozygous sire were validated by sequencing the amplicon containing the SHBG:g.27956790C>T (rs383770500) variant using an ABI 3500 Genetic Analyzer (Applied Biosystems by Life Technologies, Darmstadt, Germany) (S1 Table). PCR products for the SHBG:g.27956790C>T (rs383770500) were obtained from the primer pairs from a previous study on lethal cattle mutants [7]. Sequence analysis was done using Sequencher software, version 4.8 (Gene Codes, Ann Arbor, MI, USA). The SNV on BTA29 was amplified using the previously reported primer pairs [7].

### Statistical analysis

The ALLELE procedure of SAS/Genetics, version 9.4 (SAS Institute, Cary, NC, USA) was used to calculate polymorphism information content, heterozygosity, allelic diversity, allele and genotype frequencies and χ^2^-tests for Hardy-Weinberg-Equilibrium for the genotyped SNVs. The software OPTI-MATE, version 3.88 [8] was used to calculate the proportion of Montbéliarde and Ayrshire blood and the inbreeding coefficients for the aborted fetus from the pedigree data.

### Haplotype analysis

A total of 7 animals (paternal grandparents and the parents of the aborted fetus and its paternal half-brother and the French Montbéliarde bull (animal C) from the available pedigree) were genotyped using the Illumina bovine SNP50 Beadchip including 45,685 SNPs (Illumina, San Diego, CA, USA). Genomic DNA was isolated from EDTA-blood samples using standard methods with RBC (Red Blood Cell) lysis buffer and SE (sodium EDTA) buffer. The DNA concentration of the samples was adjusted to 50 ng/μl using the Nanodrop ND-1000 (Peqlab Biotechnology, Erlangen, Germany) and quality control was performed by gel electrophoresis using 1% agarose gels (peqGold Universal Agarose, Peqlab Biotechnology). After quality control (genotyping rate per SNP and animal >0.98), filtering for Hardy-Weinberg equilibrium (HWE) (p<0.000001) using SAS/Genetics, version 9.4 (Statistical Analysis System, SAS-Institute, Cary, NE, USA), 44,811 SNPs were left for the analysis. All SNPs of BTA29 were extracted and phased using BEAGLE, version 4 [19]. All 26 SNPs constituting the MH2 haplotype were present in this data set without missing values and haplotypes were consistent with the pedigree structure.

## Supporting Information

S1 Document Details on the MH2 haplotype linked with the T-allele of the SLC37A2:g.28879810C>T variant on BTA29. For each marker of the Illumina Bovine 50 k Beadchip, base pairs of the forward strand are denoted as A, C, G or T. The joined MH2 haplotype linked with the T-allele in Vorderwald x Montbéliarde crossbred cattle is marked with yellow.
(XLSX)

S1 Table Primer sequences used for genotyping the SHBG:g.27956790C>T mutation on bovine chromosome 19. This SNV was genotyped using a mismatch polymerase-chain-reaction-restriction fragment length polymorphism (PCR-RFLP). Primer pairs, amplicon size (AS) in base pairs (bp) and annealing temperature (AT) are given. The base T is replaced by G (in bold and brackets) in the course of the mismatch PCR in order to create a BsmFI restriction site.
(DOCX)
