# Supplementary material for: Validation of Deleterious Mutations in Vorderwald Cattle
Source: PLoS One. 2016 Jul 29;11(7):e0160013. doi: 10.1371/journal.pone.0160013 (PMC4966933; doi:10.1371/journal.pone.0160013)

**S1 Table. Primer sequences used for genotyping the SHBG:g.27956790C>T mutation on bovine chromosome 19 and validation through sequencing the amplicon with the SHBG:g.27956790C>T mutation.** This SNV was genotyped using a mismatch polymerase-chain-reaction-restriction fragment length polymorphism (PCR-RFLP). (A) Primer pairs, amplicon size (AS) in base pairs (bp) and annealing temperature (AT) are given. The base T is replaced by G (in bold and brackets) in the course of the mismatch PCR in order to create a BsmFI restriction site.

| Forward primer (5’-3’) | Reverse primer (5’-3’) | AS (bp) | AT (°C) |
| --- | --- | --- | --- |
| CCTGCTCTGCACCTCAGCAGTGGTCC**[G]**GGA | AGCTGATGGAGAGAGGGTGA | 137 | 63 |

(B) Chromatogram for the SHBG:g.27956790C>T mutation of the homozygous mutated cow and its heterozygous sire.


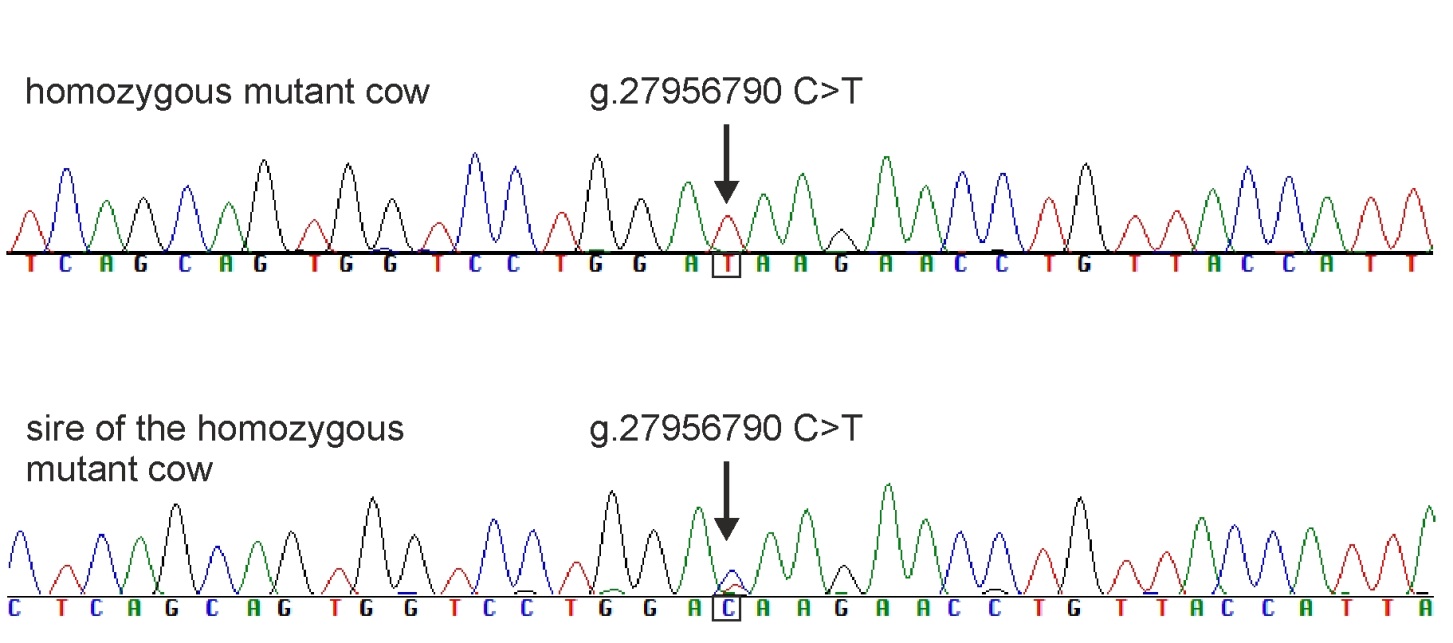

Supplement: S1 Table — This SNV was genotyped using a mismatch polymerase-chain-reaction-restriction fragment length polymorphism (PCR-RFLP). Primer pairs, amplicon size (AS) in base pairs (bp) and annealing temperature (AT) are given. The base T is replaced by G (in bold and brackets) in the course of the mismatch PCR in order to create a BsmFI restriction site. (DOCX) [file pone.0160013.s002.docx]
